# [^68^Ga]Ga-NODAGA-E[(cRGDyK)]_2_ Angiogenesis PET/MR in a Porcine Model of Chronic Myocardial Infarction

**DOI:** 10.3390/diagnostics11101807

**Published:** 2021-09-30

**Authors:** Simon Bentsen, Andreas Clemmensen, Mathias Loft, Mette Flethøj, Karina Poulsdóttir Debes, Trine Pagh Ludvigsen, Cecilie Bjørstrup Larsen, Jeppe Kirchhoff, Lisbeth Høier Olsen, Jacob Eifer Møller, Thomas Lund Andersen, Helle Hjorth Johannesen, Thomas Jespersen, Andreas Kjaer

**Affiliations:** 1 Department of Clinical Physiology, Nuclear Medicine & PET and Cluster for Molecular Imaging, Department of Biomedical Sciences, Rigshospitalet and University of Copenhagen, DK-2100 Copenhagen, Denmark; simon.bentsen.01@regionh.dk (S.B.); andreas.clemmensen@sund.ku.dk (A.C.); mloft@sund.ku.dk (M.L.); thomas.lund.andersen@regionh.dk (T.L.A.); helle.hjorth.johannesen.01@regionh.dk (H.H.J.); 2 Global Drug Discovery, Novo Nordisk A/S, DK-2760 Måløv, Denmark; MYFM@novonordisk.com (M.F.); trpl@novonordisk.com (T.P.L.); jpki@novonordisk.com (J.K.); 3 Department of Veterinary and Animal Sciences, Faculty of Health and Medical Sciences, University of Copenhagen, DK-1870 Frederiksberg C, Denmark; karina.debes@sund.ku.dk (K.P.D.); lisbeth.hoier@sund.ku.dk (L.H.O.); 4 Department of Biomedical Sciences, Faculty of Health and Medical Sciences, University of Copenhagen, DK-2200 Copenhagen, Denmark; cecilie.b.larsen@sund.ku.dk (C.B.L.); thojes@sund.ku.dk (T.J.); 5 Department of Cardiology, Copenhagen University Hospital Denmark, University of Southern Denmark, DK-5000 Odense, Denmark; jacob.moeller@regionh.dk

**Keywords:** positron emission tomography, angiogenesis, myocardial infarction, magnetic resonance imaging, late gadolinium enhancement, large animal model, cardiac imaging, PET/MRI

## Abstract

Angiogenesis is crucial in tissue repair and prevents scar tissue formation following an ischemic event such as myocardial infarction. The ischemia induces formation of new capillaries, which have high expression of integrin α_v_β_3_. [^68^Ga]Ga-NODAGA-E[(cRGDyK)]_2_ ([^68^Ga]Ga-RGD) is a promising PET-radiotracer reflecting angiogenesis by binding to integrin α_v_β_3_. A Göttingen mini-pig underwent transient catheter-induced left anterior descending artery (LAD) occlusion for 120 min, and after 8 weeks was imaged on a Siemens mMR 3T PET/MR system. A large antero-septal infarction was evident by late gadolinium enhancement (LGE) on the short axis and 2–4 chamber views. The infarcted area corresponded to the area with high [^68^Ga]Ga-RGD uptake on the fused PET/MR images, with no uptake in the healthy myocardium. To support the hypothesis that [^68^Ga]Ga-RGD uptake reflects angiogenesis, biopsies were sampled from the infarct border and healthy myocardium. Expression of α_v_β_3_ was evaluated using immunohistochemistry. The staining showed higher α_v_β_3_ expression in the capillaries of the infarct border compared to those in the healthy myocardium. These initial data confirm in vivo detection of angiogenesis using [^68^Ga]Ga-RGD PET in a translational model, which overall support the method applicability when evaluating novel cardio-protective therapies.

To evaluate angiogenesis following transient occlusion of the LAD artery, a Göttingen minipig underwent PET/MR examination [1,2]. A bolus injection of approximately 100 MBq [^68^Ga]Ga-RGD was administered through a central venous catheter. A 15 min PET acquisition was performed 30 min after the injection. After the PET acquisition, gadolinium was injected in order to examine the final infarct size.

**Figure 1:**
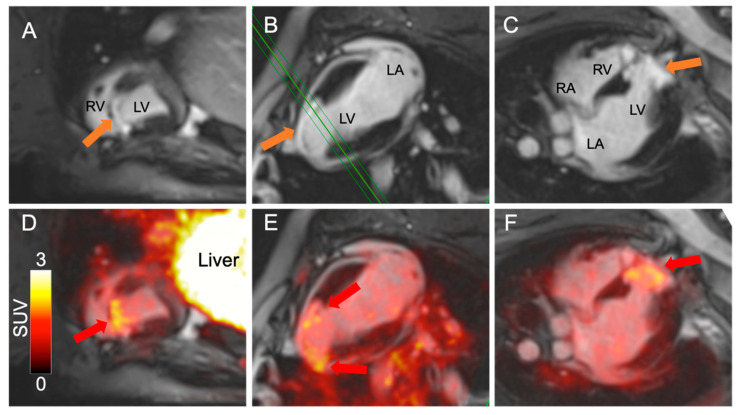
Anatomical cardiac MR images of a Göttingen minipig following gadolinium injection in short axis (**A**), two-chamber (**B**) and four-chamber view (**C**). The animal had undergone 120 min invasive LAD occlusion 8 weeks prior to the scan [3]. The green line in (**B**) illustrates the placement of the short axis slice shown in (**A**). A large antero-septal infarction is evident by late gadolinium enhancement (LGE) on all views (orange arrows). On the midventricular short axis image (**A**), the LGE appears subendocardial, while towards the apex of the heart, the infarct is transmural, indicating a large infarction. All images were obtained using a Siemens mMR 3T PET/MR system and flex body coils. The infarcted area corresponded to the area with high [^68^Ga]Ga-RGD uptake on the fused PET/MR images ((**D**–**F**), red arrows), with no [^68^Ga]Ga-RGD uptake observed in the healthy parts of the myocardium. RV: Right ventricle, LV: Left ventricle, LA: Left atrium, RA: Right atrium, SUV: Standardized uptake value.

**Figure 2:**
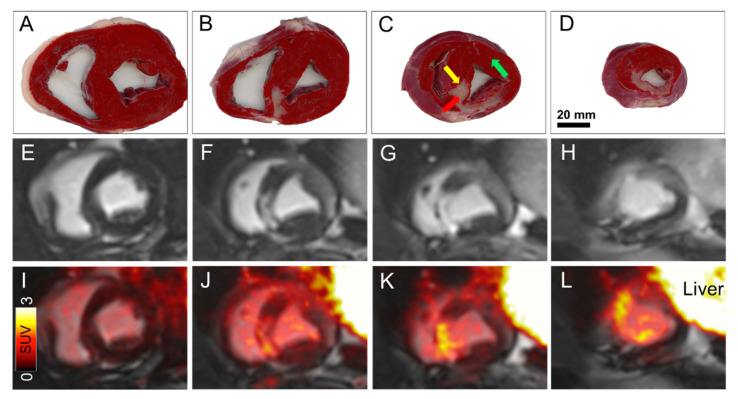
To confirm infarct severity *ex-vivo*, the excised heart was divided into 4 axial slices and stained using 1% triphenyl-tetrazolium-chloride (TTC) solution (**A**–**D**). The TTC staining in (**B**) shows a subendocardial infarction, while in (**C**,**D**), the TTC staining is transmural. The TTC staining corresponds to patterns seen by LGE on the corresponding MRI images (**E**–**H**) and fused PET/MR images (**I**–**L**). The red, yellow and green arrows in (**C**) mark the infarct zone, infarct border zone and healthy myocardium, respectively. These areas correspond to the immunohistochemical images in Figure 3. Scale bar (20 mm) is shown in (**D**) for size reference, SUV: Standardized uptake value.

This study was performed as a hypothesis-generating exploration of the evaluation of integrin α_v_β_3_ expression following transient occlusion of the LAD artery. The use of [^68^Ga]Ga-RGD as a prognostic marker for functional outcome following MI has been explored in both animal and human studies [4]. The [^68^Ga]Ga-RGD tracer in blocking studies shows a high sensitivity towards α_v_β_3_ [5], indicating the potential in future studies with a radiotracer. However, the minipig was euthanized following the PET/MR scan in order to examine histology and immunohistochemistry. This study shows promising PET images of [^68^Ga]Ga-RGD, but further studies need to be performed in order to evaluate the prognostic value of the tracer.

## Figures and Tables

**Figure 3:**
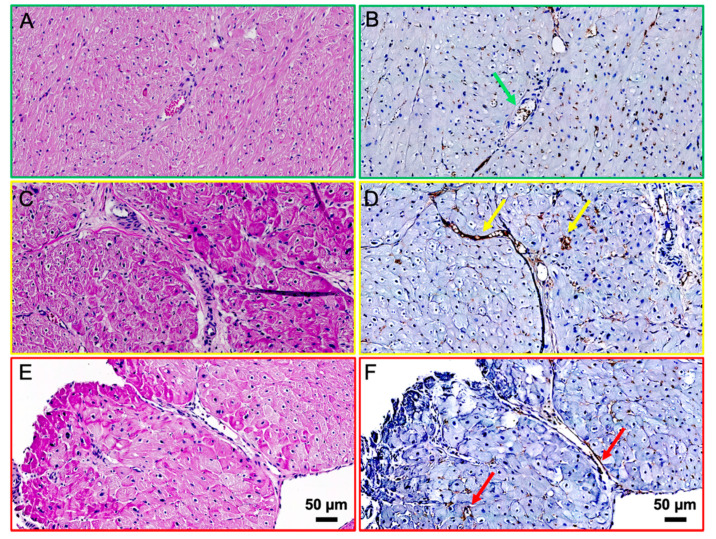
To further support the hypothesis that [^68^Ga]Ga-RGD PET uptake reflects angiogenesis, biopsies were sampled from the healthy myocardium (**A**,**B**), infarct border (**C**,**D**) and infarct area (**E**,**F**). Classic H&E staining was performed (**A**,**C**,**E**). The H&E staining of the infarcted area (**E**), shows a high degree of atypical nuclei and an amyloid-like substance, most likely collagen compared to the infarct boder zone (**C**) or healthy myocardium (**A**). The expression of α_v_β_3_ was evaluated using immunohistochemistry (**B**,**D**,**F**) (α_v_β_3_, absolute antibody; the antibody was diluted 1:100 and antigen retrieval was performed with proteinase K). The staining indicated a higher α_v_β_3_ expression in the capillaries of the infarct border ((**D**), yellow arrows), compared to those in the healthy myocardium ((**B**), green arrow) and in the infarcted area ((**F**), red arrows). Scale bar (50 µm) is shown in (**E**,**F**) for size reference.

## Data Availability

Data is contained within the article.
